# Supplementary material for: Dichocarpum hagiangense—a new species and an updated checklist of Ranunculaceae in Vietnam
Source: PeerJ. 2020 Sep 22;8:e9874. doi: 10.7717/peerj.9874 (PMC7518158; doi:10.7717/peerj.9874)
Supplement: Supplemental Information 1 — hagiangen.=D. hagiangensis; dalzie.=D. dalzielii; basila.=D. basilare; trifolio.=D. trifoliolatum; adianti.=D. adiantifolium; carina.=D. carinatum; arisanen.=D. arisanense; franche.=D. franchetii; auricula.=D. auriculatum; sutchuen.=D. sutchuense; Dicho.sp.= Dichocarpum sp. [file peerj-08-9874-s001.docx]

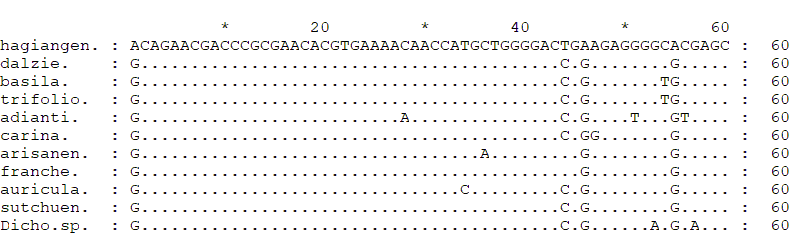

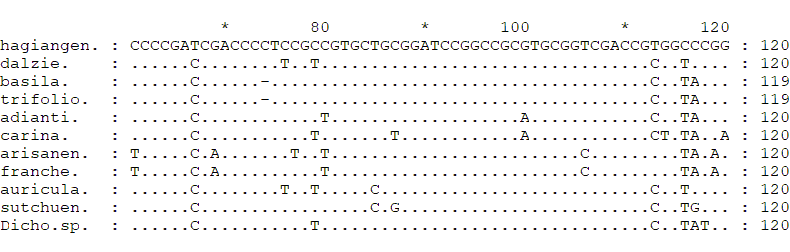

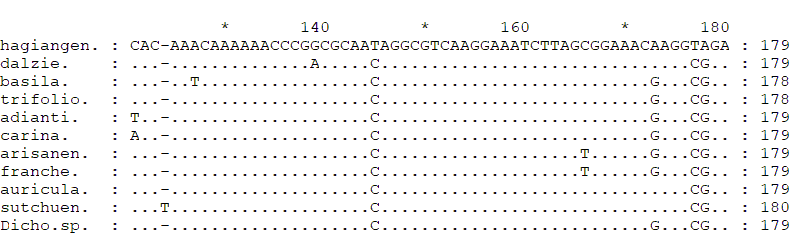

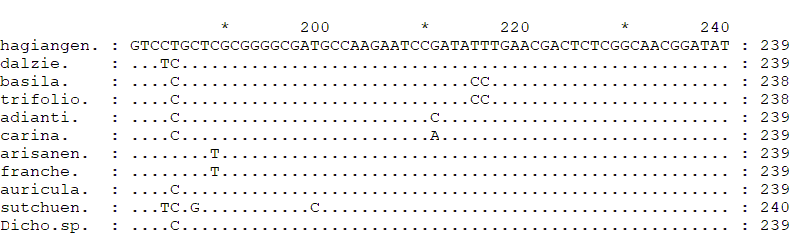

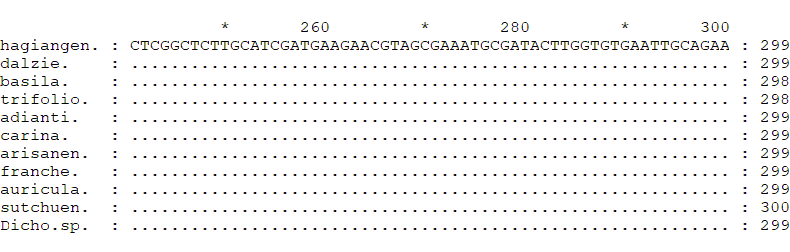

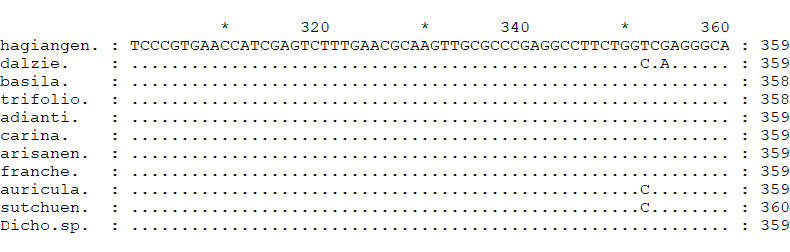

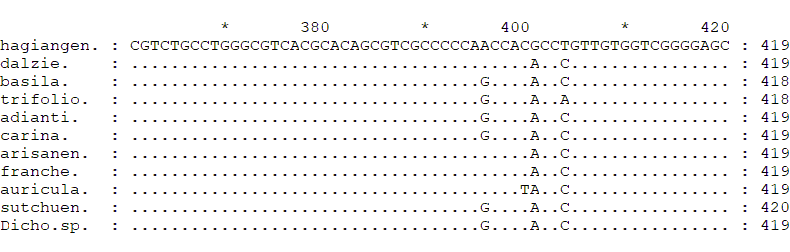

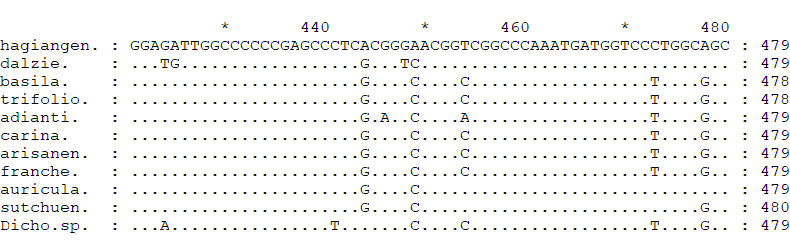

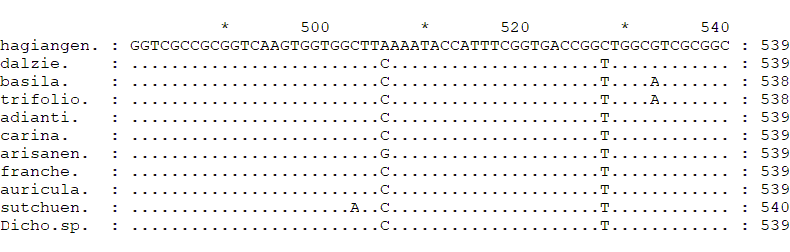

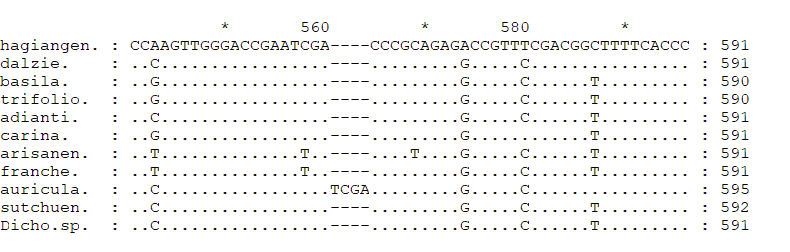
 **Supplemental.** Multiple sequence alignment of the ITS sequence of *Dichocarpum hagiangensis* with *Dichocarpum* group (hagiangen.=*D. hagiangensis*; dalzie.=*D. dalzielii*; basila.=*D. basilare*; trifolio.=*D. trifoliolatum*; adianti.=*D. adiantifolium*; carina.=*D. carinatum*; arisanen.=*D. arisanense*; franche.=*D. franchetii*; auricula.=*D. auriculatum*; sutchuen.=*D. sutchuense*; Dicho.sp.= *Dichocarpum* sp.)
